# Reply: Underutilization of Fibrinolysis Despite Timely Arrival in STEMI: A Missed Opportunity!

**DOI:** 10.1016/j.jacasi.2023.05.006

**Published:** 2023-06-20

**Authors:** Kirtipal Bhatia, Arman Qamar, Michael Hendrickson, Sameer Arora, Mohit D. Gupta

We welcome the interest of Gnanaraj and colleagues in our analysis from the NORIN-STEMI (The North Indian ST-segment elevation myocardial infarction) registry.^1^ The use of fibrinolysis was low in our study despite most patients presenting to a health care facility early after symptom onset. We attribute this to a lack of standardized treatment protocols or trained staff at health care centers where patients first made contact, many of which were primary health care centers. Additionally, prohibitive out-of-pocket expenses is a big reason for transfer to government-funded tertiary facilities, which possibly caused patients to defer medical therapy offered at the first center.

The patient population included in our study had baseline characteristics comparable to other prior Indian registries.^2^ However, because most of the patients in our cohort were referred from other health care centers, survivorship bias possibly affected the observed in-hospital mortality. This is evidenced by the fact that those receiving percutaneous coronary intervention (PCI) at less than 24 hours after symptom onset had higher mortality compared to those receiving PCI more than 72 hours after symptom onset (5% vs 3%; *P* < 0.001), and this may point to patients with less severe cases surviving naturally and subsequently receiving PCI. Gnanaraj and colleagues also highlight the high proportion of patients receiving PCI in late-presenting patients with non–ST-segment elevation myocardial infarction (STEMI). Although current guidelines recommend against PCI in asymptomatic patients, PCI is still considered reasonable among patients with persistent symptoms, with signs of heart failure, or with a large area of demonstrable viable myocardium at risk.^3^ Patients in our cohort had high rates of ventricular systolic dysfunction on presentation likely caused by delays in receiving timely reperfusion therapy. Revascularization in these patients may have been aimed at improving ventricular function. This again reinforces the unmet need of timely reperfusion for STEMI patients in India, which requires systemic reform at the national level.

A total of 310 (10%) patients were lost to follow-up at 1 year, and these patients had similar baseline characteristics compared to those who completed follow-up. Regardless, by virtue of incomplete follow-up, it is possible that 1-year mortality may be underestimated.
